# Copper sulphate impact on the antioxidant defence system of the marine bivalves *Cerastoderma edule* and *Scrobicularia plana*

**DOI:** 10.1038/s41598-019-52925-9

**Published:** 2019-11-11

**Authors:** Andreia F. Mesquita, Sérgio M. Marques, João C. Marques, Fernando J. M. Gonçalves, Ana M. M. Gonçalves

**Affiliations:** 1 Department of Biology and CESAM, University of Aveiro, 3810-193 Aveiro, Portugal; 2 MARE - Marine and Environmental Sciences Centre, Department of Life Sciences, Faculty of Sciences and Technology, University of Coimbra, 3004-517 Coimbra, Portugal

**Keywords:** Enzymes, Environmental sciences, Biomarkers

## Abstract

Anthropogenic activities, such as agriculture and industrial activities, are a main source of pollution contributing for the degradation of water quality and thus affecting the living organisms of aquatic systems. Copper is widely used at these practices being often released into the aquatic systems and may cause negative effects in its communities. This study proposes to determine the effects of copper in the antioxidant defence system of two size classes (big and small sizes) of *Scrobicularia plana* and *Cerastoderma edule*, two marine bivalve species with commercial interest. It was observed the behaviour activity of the organisms during the exposure to copper sulphate (CS) and was determined the enzymatic activities of glutathione-S-transferases (GST), glutathione reductase (GR) and glutathione peroxidase (GPx) (both selenium-dependent (SeGPx) and total (tGPx)) in the muscle tissue (foot). Lipid peroxidation (LPO) was evaluated through thiobarbituric acid reactive substances (TBARS) measurement in the foot. Changes in the behaviour and enzymatic activity were observed. Lipid peroxidation was observed at *C. edule* and *S. plana* big and small size classes, respectively, according to TBARS levels. The foot showed to be a good tissue to be used in biochemical analysis to detect the presence of toxicants.

## Introduction

Metals’ discharges in the aquatic environment are one of the major concerns in the world. Metal pollution may be from natural (e.g. volcanic activities) or anthropogenic (leaching of agricultural fields) sources^1^, leading to serious impacts on the surrounding aquatic systems and their communities. Some metals may be toxic only at great concentrations, since they are biologically essential and natural constituents of the aquatic ecosystems, being designated essential elements (e.g. copper), whereas others are toxic even at very low concentrations, being designated non-essential elements (e.g. mercury)^2^.

Copper is a well-known aquatic contaminant, due to the intense use on antifouling paints and pesticides formulations^3^. In spite of copper be an essential element, necessary to the maintenance of cellular functions^4^, acting as enzymatic cofactor and essential element of several metabolic pathways^5^, this metal may become toxic at greater concentrations and its toxicity depends on the pH and water temperature, which has increased in the recent years, with the pH decrease and temperature increase in the aquatic systems^4^. Copper toxicity can be explained by distinct mechanisms, (a) it can act establishing redox cycles, then take part in Fenton’s reaction, as catalyser agent, and consequently producing highly unstable reactive oxygen species (ROS), such as O^−^ and HO^−^; (b) the metal can interact with functional groups (e.g. carboxyl, hydroxyl, sulfhydryl) and therefore lead to its inactivation or (c) it can replace essential cofactors to the enzyme function^6–9^. Several studies revealed that the increase of the ROS production may affect many metabolic pathways, such as glycolysis, protein, fatty acids and amino acids metabolism. Furthermore, the ROS also have the ability to induce lipid peroxidation and DNA damage^10^, and ultimately can lead to the cell death. Considering the consequences in molecular processes of organisms exposed to high metal concentrations, mainly in the aquatic systems^11,12^, biomarkers are important tools and endpoints in ecotoxicology studies widely used as earlier-warning indicators of contamination. Therefore, the evaluation of a set of biomarkers, involved in the antioxidant responses, can give an indication about the antioxidant defence systems of the organisms and can be used to determine potential oxidative effects of the toxic^13,14^. Although there are numerous studies evaluating the effect of chemicals in the enzymatic activity of several organisms, none of them has described the effects of copper sulphate, in *C. edule* and *S. plana*, with a great economic interest. These species have an important ecological role, acting as link between primary producers and consumers, like fish, crustaceans or wading birds. *C. edule* lives on shallow intertidal areas showing a suspension-feeder behavior. At the other hand, *S. plana*, a deposit filter feeder, lives in subtidal and intertidal regions^15^. These bivalves species, similar to other bivalves species, are widely used in ecological and toxicological studies, since they have a large filtration ability and may accumulate high quantities of pollutants in their tissues^16–20^. Moreover, their sessile lifestyle, the ease of sampling collection, maintenance in laboratory and sensitivity to chemicals make them good standard species, being also considered good bioindicators^21^.

By this, the present work aims to determine: (a) the oxidative stress response of Glutathione S-Transferases (GST), Glutathione Reductase (GR) and Glutathione Peroxidase (GPx); (b) the thiobarbituric acid reactive species (TBARS) levels, to verify the occurrence or not of lipid peroxidation (LPO), in the muscle tissue (foot) of big and small size classes of both marine species, *C. edule* and *S. plana*, under copper sulphate contamination.

## Materials and Methods

### Study area and field collection

**Figure 1:**
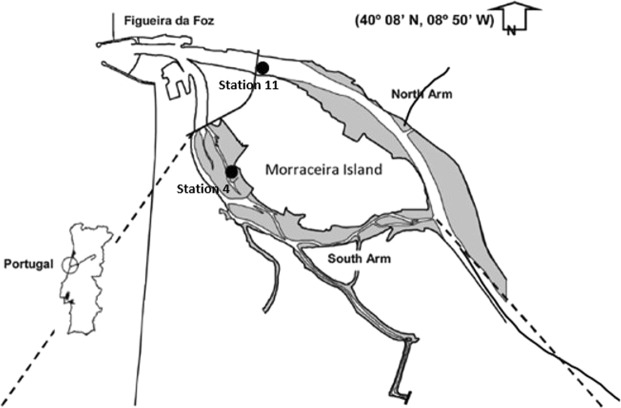
Mondego estuary and sampling areas location (Station 4 and Station 11) within the estuary (The authors acknowledge Dr. Tiago Verdelhos to give permission for the use of Fig. 1).

The Mondego estuary is a small estuarine system, situated in the Portuguese Western coast, near Figueira da Foz city (40°08′N, 8°50′W), covering about 3.4 km^2^, with around 8 km of extension. The north and south arms of estuary represent two distinct subsystems with different hydrodynamic characteristics, and divided by the Morraceira Island. Mondego estuary has an important socio-economic role, given the variety of resources and services provided to the population. Consequently, this system is under an intensive anthropogenic pressure (copper concentrations of 0.74 to 1.4 µg/L were reported along of the estuary^22^), with eutrophication stress mainly due to the excessive nutrients inputs from the Pranto River. The organisms were collected in the two arms (Fig. 1) in the morning at the end of the spring, with dredge support and transported to the lab in cold boxes with field water. The sampling was done in the sites where the species were more abundant at the Mondego estuary. There is not a reference site to be considered as the anthropogenic pressures that the estuarine system is subjected. Water (from the surface and from the bottom) and sediment from the sampling sites were also collected to copper quantification in the laboratory.

### Laboratory procedures and bivalve bioassays

At the laboratory, was selected 10 bigger and 10 smaller organisms from each species, not exposed to any laboratory procedure and were measured some parameters (the shell length, the total weight, the tissue weight and the foot weight) to determine the condition indices. The foot of each individual was stored at −80 °C until further biochemical analysis.

The other sampled organisms were subjected to a depuration period, over 48 hours, without food, in seawater at 20 of salinity, previous filtrated. After this time, the individuals of each species were maintained in different aquaria according to the size class: small size class (S) – body size of *C. edule* = 1.97 (1.64; 2.37) cm; body size of *S. plana* = 3.47 (2.77; 4.07) cm; big size class (B) - body size to *C. edule* = 2.45 (2.24; 2.72) cm; body size to *S. plana* = 4.20 (3.82; 4.87) cm.

With the purpose to evaluate the effect of copper sulphate on the antioxidant defence system but also in the fatty acids profile^23^ of two bivalve species, two experiments were carried out simultaneously. *C. edule* and *S. plana* were exposed to a range of 7 concentrations between 0.35 mg/L and 2.1 mg/L and between 1.0 mg/L and 4.0 mg/L, respectively, plus the negative control. *S. plana* was submitted to higher concentrations than *C. edule*, since at previous tests the peppery furrow shell achieved a survival rate of 100% to all treatments and consequently was not possible to calculate the lethal concentration. Each treatment was composed by ten replicates, one organism per replicate, with a volume of 1 L and 0.5 L of medium at each vessel to big and small size classes respectively, to both species. A frozen commercial mix of microalgae and rotifers (by Ocean Nutrition) was used to feed the inviduals daily, and the medium changed every two days. Bioassays occurred during 96 hours, under a 12 h^L^: 12h^D^ photoperiod, controlled temperature (20 ± 2 °C), with sea water medium, previously filtrated, and with a salinity of 20 psu (considered the optimum salinity to both species)^15^, with aeration system. At the end of the exposure period, all survival organisms were dissected, measured and weighted and their condition indices were evaluated, so some organisms were randomly selected to the fatty acids analysis described by Mesquita *et al*.^23^ and the others used to the evaluation of the antioxidant defence system.

### Mortality and behaviour activity

Individuals of big and small size classes of *C. edule* and *S. plana* were observed daily to analyse mortality and behavioural conditions, being evaluated the siphon activity, valves condition, and the organism behaviour during feeding. Mortality (inability to close the valves upon mechanical stimulus) was observed and registered every 12h, and dead individuals removed from the flask, allowing to calculate the lethal concentration to both size classes of each species.

Behaviour activity was determined considering three main behavioural characters which were recorded as 0/1 corresponding to its absence/presence. It was considered siphon activity, related to feeding and excretion activities (0 means no siphon activity and 1 means observed or signs of siphon activity); valves activity related to the ability of the valves to close (0 means no ability and 1 means ability to close); and reaction to mechanical stimulus or perturbation, such as light or touch, e.g. siphon, valves, foot (0 means no or slow reaction and 1 means instant reaction)^24^.

Each behavioural character was estimated through the quotient between the organisms that exhibited activity and the total organisms (e.g. Siphon activity = *n* Observed siphon activity/*n* Total individuals), where “*n*” means the number of individuals in each treatment; range from 0 to 1. Additionally, the activity in each treatment was determined as the sum of the three traits (Siphon activity, Valves activity and Reaction), range from 0 to 3. Lastly, an activity index for the entire assay was estimated as:

$${\rm{Total}}\,{\rm{Activity}}=\frac{{\rm{Activity}}\,(\mathrm{24h})+{\rm{Activity}}\,(\mathrm{48h})+{\rm{Activity}}\,(\mathrm{72h})+{\rm{Activity}}\,(\mathrm{96h})}{4}$$

according to Verdelhos *et al*.^15^.

### Copper quantification

Water samples were collected from the both sampling sites and the different treatments of the bioassays. The samples were sampled to plastic bottles and acidified with nitric acid Pro Analysis MERCK® (65%) to a pH bellow 2, to prevent metal adsorption, according to the protocol described by Marques *et al*.^25^, to then be analysed, at the laboratory, to quantify copper concentrations. The tissues were dried at 105 °C by about 24 hours and then weighted and digested in closed falcon tubes with 3 ml HNO_3_ (Pro Analysis MERCK®, 65%), at 60 °C, on the oven, by about 8 hours to copper quantification. After this step, 0.5 ml of hydrogen peroxide (Suprapur MERCK®, 30%) was added to each sample to prevent any type of organic matter, and the samples were put on the oven by 2 hours. The volume was completed to 5 ml per sample with Milli-Q® water (18.2 ῼ), according to Marques *et al*.^25^.

To quantify the copper concentration on the sediment samples, sediment was sieved and homogenized, with a sieve below 2 mm. About 2 grams (dry weigh) of each replicate was weighted and digested with *Aqua Regia* (3 ml of hydrochloric acid ANALAR NORMAPUR, 37% + 1 ml of nitric acid Pro Analysis MERCK®, 65%) in Teflon beakers. Each sample was slowly heated on a hotplate at 100 °C until to dry. Then 10 ml of nitric acid (4N) was added and the solution was filtered through 0.45 µm to remove the particles, following the protocol described by Pereira *et al*.^26^. The effective copper concentration was determined using flame atomic absorption spectrophotometry (ICE3000 Series AA Spectometer– Thermo Scientific), being the limit of quantification for undiluted samples of 10 µg/L.

Copper quantification was performed to the tissues of the organisms from the field and under bioassays conditions, to the water samples collected at the field and used to the bioassays, and to the sediment collected at the field. The sediment was not used to the experiment, according to the results of previous works of our research team^15^.

### Biomarkers analysis

The oxidative stress responses were evaluated in the muscle tissue (foot) by the determination of GPx (total and selenium dependent), GR and GSTs activity and the lipid peroxidation by the TBARS measurement. Muscle tissue was homogenized in ice-cold phosphate buffer (50 mM, pH = 7.0 with 0.1% Triton X-100). Afterwards, the homogenized was centrifuged at 15000 G during 10 minutes and the supernatants divided into five aliquots (one for each determination – GSTs, GR, GPx, TBARs) and one to protein quantification. The aliquots were stored at −80 °C for subsequent determination. At the end, all biomarkers were expressed in function of the protein content of each corresponding sample. Moreover to statistical aims and to reduce the biochemical determination variability, it was considered the average biomarker value of six organisms by treatment.

#### GSTs

GSTs (EC 2.5.1.18) activity was evaluated through spectrometry, according to Habig *et al*.^27^, and it was used a phosphate buffer (0.1 M), pH = 6.5. GST catalyzes the conjugation of substrate 1-chloro-2, 4-dinitobenzene (CDNB) with the reduced glutathione (GSH), originating a thioeter (molecular extinction coefficient of 9.6 mM^−1^cm^−1^), that can be monitored by the absorbance increase at 340 nm (with a continuous read during 5 minutes). Enzymatic activity was determined in quadruplicate and the results were expressed in nmol of substrate hydrolyzed per minute per mg of sample protein.

#### GR

GR (EC 1.8.1.7) activity was assayed by spectrometry, according to protocols of Carlberg and Mannervik (1985)^28^, and it was used a phosphate buffer (200 mM) with EDTA (2 mM) and a pH = 7.0. GR involved in the NADPH oxidation was following at a wavelength of 340 nm (molecular extinction coefficient of 6.22 mM^−1^cm^−1^) by 5 minutes. Enzymatic activity was determined in quadruplicate and the results were expressed in nmol of NADPH oxidized per min per mg of sample protein.

#### GPx

GPx (EC 1.11.1.9) activity was determined according to Flohé and Günzler (1984)^29^, using a phosphate buffer (100 mM), with pH = 7.0, following the NADPH oxidation at 340 nm (molecular extinction coefficient of 6.22 mM^−1^cm^−1^) by 5 minutes, when oxidized glutathione (GSSG) is reduced back to the reduced form by the reductase glutathione. The GPx activity was evaluated using two independent substrates, the hydrogen peroxide (0.255 mM) to the glutathione peroxidase selenium-dependent and the cumene hydroperoxide (0.7 mM) to the total GPx measurement. Enzymatic activity was determined in quadruplicate and the results were expressed in nmol of substrate hydrolyzed per minute per mg of sample protein.

#### TBARS

The lipid peroxidation was evaluated by the TBARS quantification, according to Buege and Aust (1978)^30^. The TBARS levels were measured by spectrometry, was determined in duplicate at 535 nm (molecular extinction coefficient of 1.56 × 10^5^ mM^−1^cm^−1^) in a single read, based on the reaction of lipid peroxidation by-products, with 2-thiobarbituric acid (TBA), therefore the results were expressed in nmol per mg of protein.

#### Total protein concentrations

The total concentration of protein of each sample was determined by spectrometry at 595 nm, according to Bradford method^31^ adapted to the microplate. The Bradford method depends on the link of the dye Coomassie Blue G-250 to proteins, and the protein concentration can be estimated by comparison with a standard solution of γ-bovine globulin.

### Statistical analysis

The statistical analysis was performed using the SPSS Statistics 20 software. To evaluate the existence of statistical significant differences between the organisms exposed at different copper concentrations, regarding to antioxidant enzymes and TBARs levels, the analysis of variance (ANOVA) assumptions was checked and a one-way ANOVA was carried out, followed by a Tukey’s test to compare all treatments (including the organisms exposed to copper sulphate at the bioassays, and the organisms from the field and after depuration period). To reject de null hypothesis was considered a level of significance less than 0.05, and the significant differences between all conditions and treatments were marked by different letters.

## Results

### Exposure to copper sulphate

#### Mortality

Considering the LC values, already described in Mesquita *et al*. (2018), for both species, *C. edule* exhibited less tolerance than *S. plana* to copper sulphate to both size classes. Moreover, the bigger organisms showed to be more sensitive than the smaller ones, regarding both bivalve species (Online Resource 1).

#### Behaviour activity

**Figure 2:**
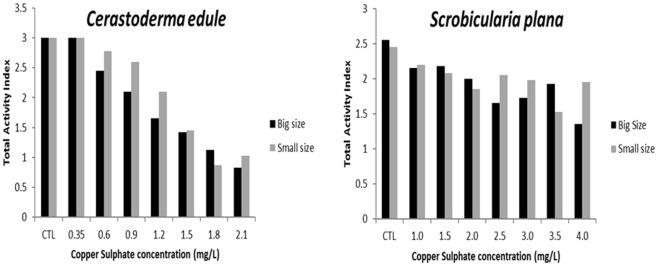
Behavioural activity, measured through the total activity index, along with copper sulphate concentrations (mg/L) to big and small size classes of the bivalves *Cerastoderma edule* and *Scrobicularia plana* (small size class is represented by light grey bars and black bars represent the big size class).

Behaviour was evaluated observing the organisms’ activity during the experiment. The absence/presence of 3 behavioural characters (siphon activity, valves activity and reaction) was registered and integrated into an activity index to both species and size classes. In the case of *C. edule*, it was observed a gradual decrease of the activity index with the increase of the copper sulphate concentrations, being these differences statistically significant at the highest concentrations (1.5 mg/L, 1.8 mg/L and 2.1 mg/L) to big and small size classes; individuals of small size class exhibited a bigger activity index than the big size class of organisms at almost all treatments, except to the exposure at the lowest concentration (0.35 mg/L) and the treatment of 1.8 mg/L of the compound (Fig. 2). However, in the case of *S. plana*, it was not possible to observe a so defined pattern. Regarding big size class of organisms, it was observed a decrease in the total activity index at the four first concentrations of copper sulphate, with an increase of total activity index at the fifth and sixth concentrations and a new decrease in the last concentration; significant decreases comparing with the control treatment were observed only at the concentrations of 2.5 mg/L, 3.0 mg/L and 4.0 mg/L. On the other hand, at the small size class of organisms, it was observed a decrease of the total activity index at the three first concentrations of the compound, with an increase at the fourth concentration, followed by a decrease at the next two concentrations and an increase at the last concentration of the chemical. Furthermore, significant differences comparing with the control treatment were not observed. The larger organisms exhibited a greater total activity index than the smaller organisms, to the concentrations of 1.5 mg/L, 2.0 mg/L and 3.5 mg/L of copper sulphate (Fig. 2).

### Copper quantification

The analysis to the copper quantification are expressed in Table 1. Regarding to the samples from the field, only the copper concentration in the sediment was quantified, since was not possible to quantify copper at the water collected at the surface and at the bottom either at the organisms’ tissues due to the detection limit of the equipment together to the insufficient organisms’ biomass in the cause of the tissues’ analysis. The same constrains occurred to the tissues’ samples from the bioassays, being only possible the quantification of the copper concentration at the medium. Due to the number of biomarkers analysis to be conducted, and the biomass needed to each one, the quantity to each analysis was quantified and rigorously divided.

**Table 1:** Copper concentrations quantified at the sediment (in grey) and water from the field and at the water used on the bioassays.

			Field		
	Site		Copper Concentration Quantified (mg/Kg)		
Sediment	North Arm		9.13		
	South Arm		8.13		
	Site		Copper Concentration Quantified (mg/L)		
Water	North Arm		<0.1		
	South Arm		<0.1		
			Bioassays		
	**Species**	**Treatments**	**Copper Sulphate Concentration Estimated (mg/L)**	**Copper Concentration Estimated (mg/L)**	**Copper Concentration Quantified (mg/L)**
Medium	*Cerastoderma edule bioassay*	CTL	0.00	0.000	0.000
		C1	0.35	0.089	0.100
		C2	0.6	0.153	0.180
		C3	0.9	0.229	0.275
		C4	1.2	0.305	0.374
		C5	1.5	0.382	0.403
		C6	1.8	0.458	0.422
		C7	2.1	0.509	0.477
	*Scrobicularia plana bioassay*	CTL	0.00	0.000	0.000
		C1	1.0	0.254	0.339
		C2	1.5	0.382	0.403
		C3	2.0	0.509	0.477
		C4	2.5	0.636	0.690
		C5	3.0	0.763	0.823
		C6	3.5	0.891	1.003
		C7	4.0	1.018	1.206

### Biomarkers

The analysis of enzymatic responses was performed only to the organisms from the field, depuration time and exposed to concentrations, when the mortality rate was lower than 50% (*C. edule*_Big_: CTL, 0.35 mg/L and 0.60 mg/L; *C. edule*_Small_: CTL, 0.35 mg/L, 0.60 mg/L and 0.90 mg/L; *S. plana*_Big_: CTL, 1.00 mg/L, 1.50 mg/L, 2.00 mg/L; *S. plana*_Small_: CTL, 1.00 mg/L, 1.50 mg/L, 2.00 mg/L, 2.50 mg/L, 3.00 mg/L and 4.00 mg/L).

**Figure 3:**
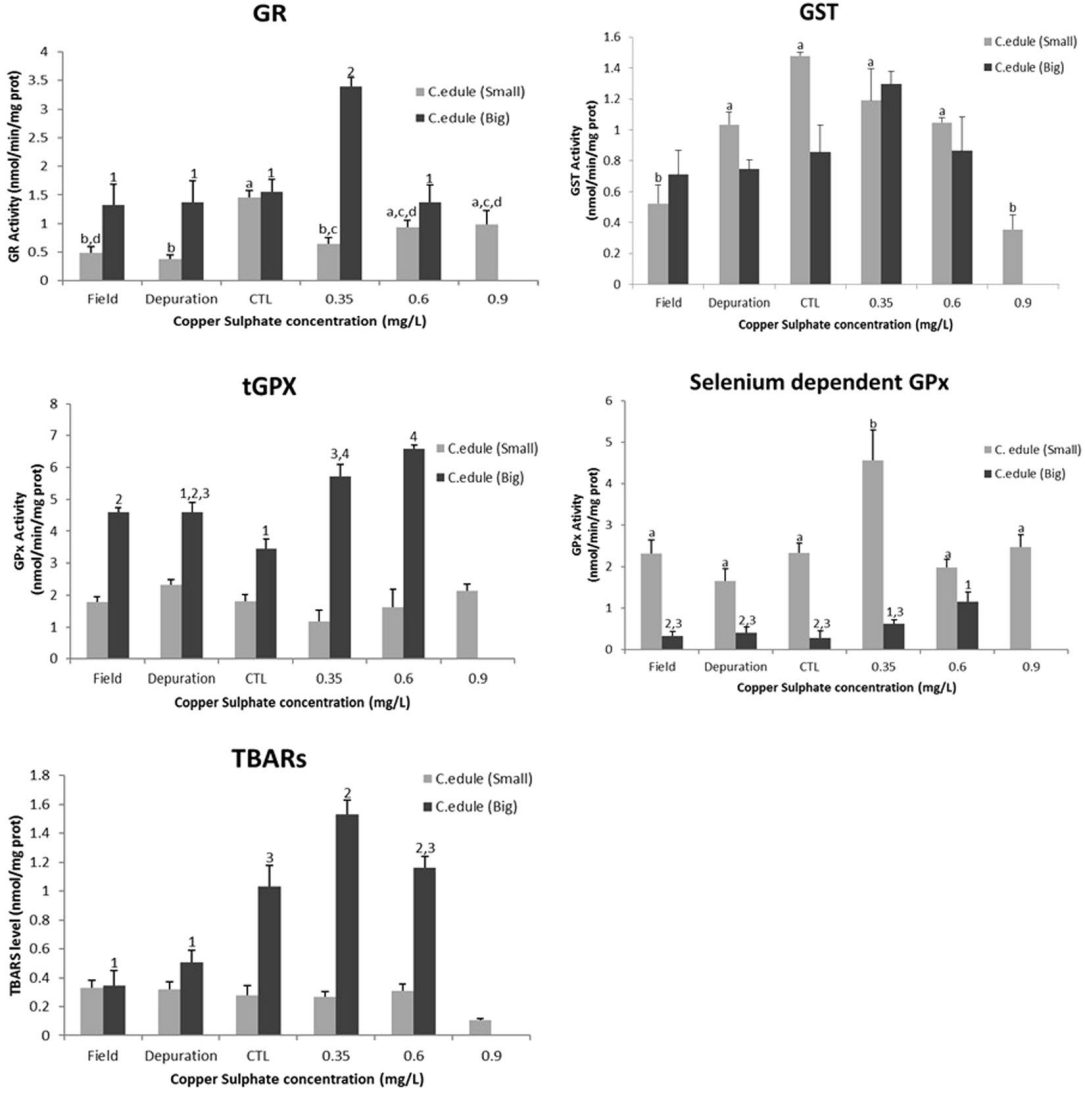
Glutathione S –transferase (GST), glutathione reductase (GR); total-glutathione peroxidase (tGPx), selenium dependent- glutathione peroxidase (SeGPx) activities and Tiobarbituric reactive species (TBARS) levels, in the foot of *Cerastoderma edule* (big and small size classes) from the field, after depuration period, control and copper sulphate treatments under laboratory conditions. Error bars represent standard error and the similar enzymatic activity between the conditions (statistically significant difference to p value < 0.05) are express by equal letters (small size class) and numbers (big size class).

Biomarkers analysis showed significant differences, comparing the control treatment, for the two species and size classes, with after the exposure to copper sulphate. In the case of *C. edule* (Fig. 3), regarding big size class, at GSTs analysis were not observed significant differences among the treatments, only it was observed a little activity increase at 0.35 mg/L of copper sulphate, but no statistically significant. GR only exhibited significant differences, comparing with the control treatment, at the first concentration (0.35 mg/L) of the chemical. Moreover, it was observed a slightly increase of the selenium dependent GPx activity, with significant differences at 0.6 mg/L of copper sulphate when compared to the control but without significant difference when compared to 0.35 mg/L, with total GPx also presenting an increase at the activity with the concentration gradient, being observed significant differences at all conditions, except to the organisms under depuration period, when compared to the control treatment. TBARs levels were also evaluated and the organisms exposed to 0.35 mg/L of copper sulphate showed significant differences, revealing more lipid peroxidation on the organisms exposed at 0.35 mg/L of copper sulphate. Regarding to small size class, the organisms from the field and exposed to the higher concentration of copper sulphate (0.9 mg/L) exhibited significant differences in the GSTs activity, comparing to the control. Additionally, it was possible to observe a decrease of the GSTs activity with the increase of copper sulphate concentration, with a significant decrease at the concentration of 0.9 mg/L, GR activity presented significant differences, with the control, at the organisms from the field, under depuration period and exposed at the first concentration of copper sulphate, observing inhibition of the activity. Furthermore, at total GPx were not observed significant differences at any treatment, nevertheless selenium dependent GPx revealed significant differences only to the organisms exposed to 0.35 mg/L of copper sulphate, where it was exhibited a raise in the activity. Still, it was not observed significant differences at the TBARS.

**Figure 4:**
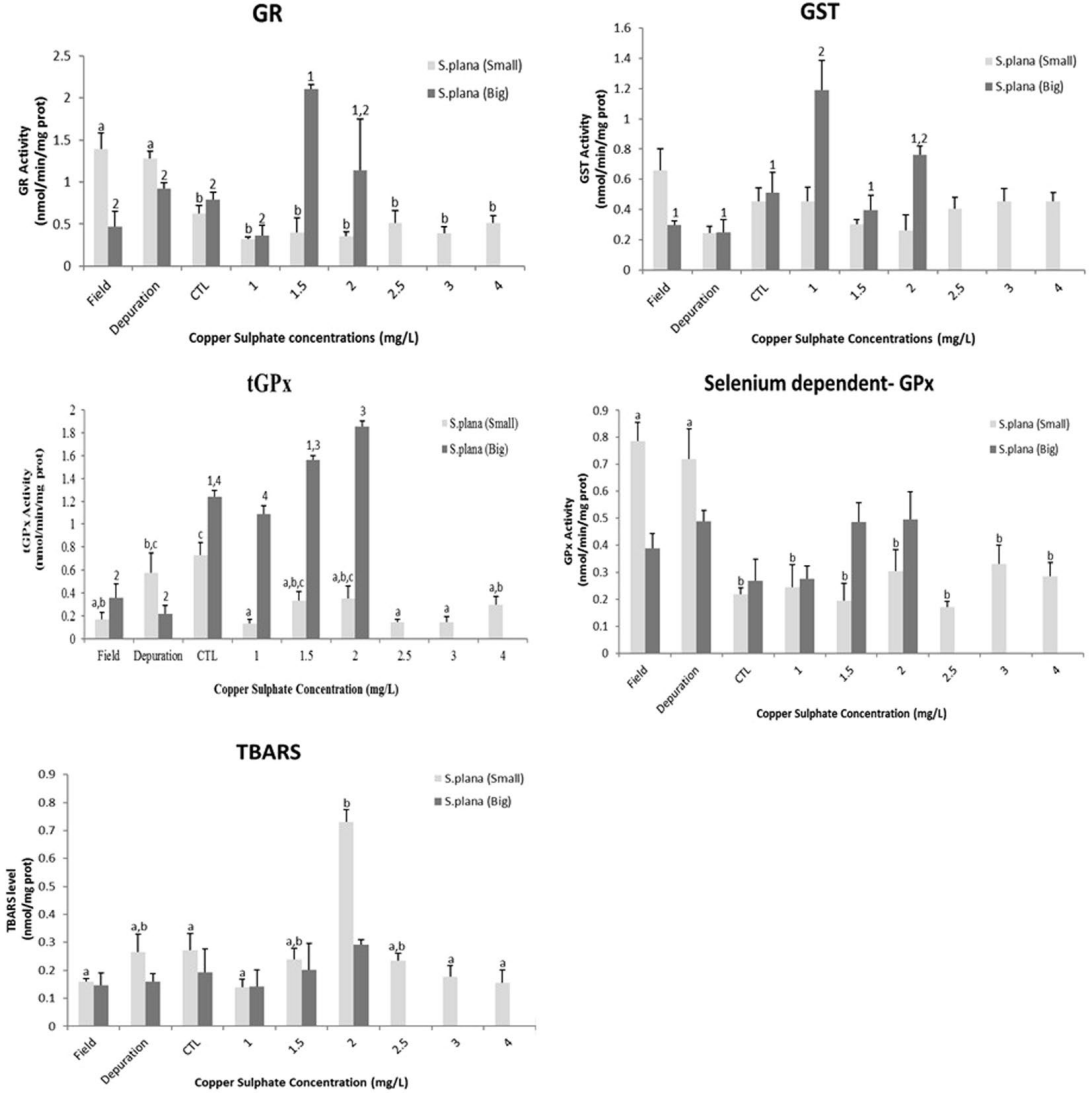
Glutathione S –transferase (GST), glutathione reductase (GR); total-glutathione peroxidase (tGPx), selenium dependent- glutathione peroxidase (SeGPx) activities and Tiobarbituric reactive species (TBARS) levels, in the foot of *Scrobicularia plana* (big and small size classes) from the field, after depuration period, control and copper sulphate treatments under laboratory conditions. Error bars represent standard error and the similar enzymatic activity between the conditions (statistically significant difference to p value < 0.05) are express by equal letters (small size class) and numbers (big size class).

On the other hand, considering *S. plana* (Fig. 4), organisms of big size class showed a significant increase of the GST activity at the concentration of 1.0 mg/L of copper sulphate. At GR activity, at the concentration of 1.5 mg/L of the compound, it was also observed significant differences. In the total GPx activity at the organisms from the field, under depuration period and exposed to 2.0 mg/L of copper sulphate. Moreover, it was noticeable a trend of gradual increase of the selenium dependent GPx activity and TBARS levels, along of the concentration range with no observed significant differences. The small size class of organisms did not show significant differences to GSTs activity at any treatments, only showing a little inhibition up to the fourth concentration of the toxic; GR and selenium dependent GPx activities were significantly higher at the organisms from the field and under depuration period; nonetheless total GPx activity presented significant inhibition to all conditions, whereas the organisms exposed to 2 mg/L of copper sulphate showed a significant increase at TBARs levels, suggesting the occurrence of lipid peroxidation.

## Discussion

This work highlights ecotoxicological and biochemical effects of copper sulphate in two bivalve species with the small size organisms of the two species showing a higher tolerance to the pollutant than the big size class. Moreover, the common cockle individuals of the two size classes exhibited a less tolerance to the compound than the peppery furrow shell organisms.

Even enzyme activity studies are usually done considering the whole organism, the digestive gland or the gills, this study just analysed the foot tissue. The utilization of the foot tissue showed efficient in the copper sulphate effects determination and allows us to economise the resources, since the chemicals and technics used to the enzymatic analysis are quite expensive and using a less amount of tissue it is possible to reduce the costs and also to obtain the right results. Moreover, a great lipid content are stored in the muscle tissue (foot) and the utilization of this tissue to determination of the enzymatic parameters it also allows us to understand the enzymatic causes of the lipid peroxidation occurrence in this tissue.

Many studies report the copper sulphate and copper as being toxic to bivalve species, as compiled on Table 2. A LC_50_ of 3.990 mg/L of copper sulphate was reported by Satyaparameshwar *et al*.^32^ to *Lamellidens marginalis*, after an exposure of 72 h. When compared with the organisms in this study, *L. marginalis* shows to be more tolerant to copper sulphate than *C. edule* (LC_50_B = 0.818 (0.595; 0.987) mg/L; LC_50_S = 1.129 (0.968; 1.289) mg/L), as well as the large organisms of *S. plana* (LC_50_ = 2.563 (2.229; 2.903) mg/L). A LC_50_ of 18.9 (10.0–31.1) µg/L to *Anodonta anatina*, a LC_50_ of 29.3 (25.4–34.0) µg/L to *Pseudanodonta complanata* and a LC_50_ of 19.0 (16.2–22.0) µg/L to *Unio tumidus* was stated by Kováts *et al*.^33^ when organisms were exposed to copper. Sousa *et al*.^34^ exposed the gastropod *Nassarius reticulatus* to copper during 96 hours, reporting a LC_50_ of 58.84 (35.45–82.29) µg/L. So, considering the copper percentage on copper sulphate at the range of concentrations here studied, our work highlights that *C. edule* and *S. plana* show a higher tolerance to copper action than other molluscs reported by other researchers. *Daphnia magna*, a standard organism in ecotoxicology, was exposed by 96 hours to copper sulphate and exhibited a LC_50_ = 0.0826 (0.0823; 0.0829) mg/L^35^, showing to be more sensitive to the compound action than the species here studied.

**Table 2:** Summary of copper sulphate effects on different species, reported by previous researches, and comparison with our results.

	Species	Exposure Time	Chemical	Concentration	Effect	Author
Lethal Effects	*Cerastoderma edule*	96 hours	Copper sulphate	LC_50 (Big size)_ = 0.818 (0.595–0.987) mg/L LC_50 (Small size)_ = 1.129 (0.968–1.289) mg/L	Lethality	23
	*Scrobicularia plana*	96 hours	Copper sulphate	LC_50 (Big size)_ = 2.563 (2.229–2.903) mg/L LC_50 (Small size)_ = 4.705 (3.540–12.292) mg/L	Lethality	23
	*Lamellidens marginalis*	72 hours	Copper sulphate	LC_50_ = 3.990 mg/L	Lethality	32
	*Anodonta anatine* *Pseudanodonta complanata* *Unio tumidos*	48 hours	Copper	LC_50_ = 18.9 (10.0–31.1) µg/L LC_50_ = 29.3 (25.4–34.0) µg/L LC_50_ = 19.0 (16.2–22.0) µg/L	Lethality	33
	*Nassarius reticulatus*	96 hours	Copper	LC_50_ = 58.84 (35.45–82.29) µg/L	Lethality	34
	*Daphnia magna*	96 hours	Copper sulphate	LC_50_ = 0.0826 (0.0823–0.0829) mg/L	Lethality	35
Effects on Enzymatic Activity	*Cerastoderma edule*	96 hours	Copper sulphate	0.35–0.9 mg/L	Increase of GR, GST, GPx activities and TBARS levels, to the big size class, at the lower concentration. Decrease of GR activity to small size class at 0.35 mg/L and of GST activity along of the concentration range, being statistically significant at 0.9 mg/L.	Our results
	*Scrobicularia plana*	96 hours	Copper sulphate	1.0 – 4.0 mg/L	Increase of TBARS levels on both size classes at 2.0 mg/L. Increase of tGPx activity to small size class, at all concentrations.	Our results
	*Scrobicularia plana*	16 days	Nanoparticles of copper oxid	10 µg/L	Increase of GST; no changes in TBARS levels	49
	*Chlamys farreri*	96 hours	Copper	3 µg/L	No effects on GST and GPx activities	9
	*Perna perna*	72 hours	Copper sulphate	40 µg/L	Decrease of GSH levels, with no significant changes on GST and GPx activities	50
	*Mytilus galloprovincialis*	72 hours	Copper	40 µg/L	Decrease of GSH levels	51
	*Mytilus galloprovincialis*	6 days	Copper	40 µg/L	Increase of lipid peroxidation	53–54
	*Ruditapes decussatus*	28 days	Copper	2.5 µg/L	Increase of lipid peroxidation	55

The behavioural activity results showed a decrease of the activity index along the copper sulphate concentrations to both size classes of *C. edule*, with the smaller organisms exhibiting often major activity index than the bigger organisms, except to the chemical concentrations 0.35 mg/L and 1.8 mg/L and the control treatment. Regarding to *S. plana*, this pattern was not observed. Moreover, the small size class organisms exhibited greater activity index than the bigger ones at the copper sulphate concentrations of 1.00 mg/L, 2.50 mg/L, 3.00 mg/L and 4.00 mg/L.

Several studies showed that the exposure to the metal leads to the increase of reactive oxygen species (ROS) production in the cells^13,36^ that can cause several cellular damages. Accordingly, copper toxicity may be due to generation of ROS via Fenton or Haber-Weiss reactions^37,38^. So, regarding the large organisms of the two bivalve species, it was detected the activation of the enzymatic antioxidant defence system, suggesting an attempt of detoxification. The simultaneous increase of GR, GSTs and GPx activity, to big size class of *C. edule*, at 0.35 mg/L of copper sulphate, may suggest the GSH increase, which in the presence of metals can be synthesized, helping to detoxification. However, the detoxification process did not prove effective, how is suggested by the increase of TBARS level, revealing the possible increase in lipid peroxidation on *C. edule* and *S. plana* organisms at 0.35 mg/L and 2.0 mg/L, respectively. So, once again *S. plana* seems to display more tolerance to copper sulphate than *C. edule*, since the lipid peroxidation occurs to a higher concentration. In the other hand, regarding the small size classes, at *C. edule* was verified a statistically significant inhibition of GR activity at 0.35 mg/L of compound and a declining trend of the tGPx activity at the concentration, although not statistically significant. Moreover, the decrease of the GST activity along the concentrations, being this inhibition statistically significant at the highest concentration, suggests the reduction of the GSH availability. The SeGPx is part of a second defence line on the antioxidant system^39^ and was not inhibited, contrariwise it increased significantly at the concentration of 0.35 mg/L, which may explain the efficiency in the fight against the lipid peroxidation, once it was not observed significant changes at the TBARS levels. *S. plana* only presented a statistically significant inhibition to tGPx activity to all concentrations, with the others antioxidants enzymes do not exhibit significant changes compared to the control situation. Then the antioxidant defence system showed inefficient to protect the organisms against ROS action, suggested by the increase of lipid peroxidation at the concentration of 2.0 mg/L of copper sulphate, given the increase of TBARS levels at this concentration.

Although different responses were observed between the control and the organisms from the field and after the depuration period, these differences may be not due only to the action of copper sulphate, but also to other stress factors, such as variations of temperature, salinity, diet quality, not controlled in the field and absence of the food during the depuration period. In the field, the organisms are under the action of several contaminants, since in the Mondego estuary was reported the presence of Hg, Cu, Cd, Cr, and Zn, that may become from anthropogenic activities, such as diffuse sources and discharges of the Pranto tributary and higher levels of Fe, maybe associated to precipitation of Fe oxides^40^, which also affect the antioxidant defence system of aquatic organisms^6^. Changes between the organisms from the field and under the depuration time, regarding to the organisms submitted to the control treatment are quite expected, since a recent study by Gonçalves *et al*.^41^, evaluating the effect of salinity on the antioxidant system of the studied species, *C. edule* and *S. plana*, showed alterations at the enzymatic activity level with the salinity changes. Similar results were observed by others authors to others species, such as to *Venerupis corrugata*^42^, *Litopenaeus vannamei*^43^, and also to *S. plana*^44^. On the other hand, the depuration process, often performed in sterile sea water, during 24 to 72 hours^45^ is also pointed out as able to induce traumatic and physiologic stress, leading to debility and death of the organisms^46^. Ruano *et al*.^47^ observed that depuration process, effectively induces stress at biochemical level on the bivalves’ species, with consequences on the condition index and nutritional level, also observed in this work.

Several studies evaluated the effects of copper on the antioxidant system of bivalve species, being found different and transitory responses on mussels exposed to metals in the field and under laboratory conditions^48^. Buffet *et al*.^49^ reported to *S. plana*, exposed to nanoparticles of copper oxid (CuONP) during 16 days, a significant increase of GST activity in the soft tissues, trend also observed in this work to the big size class of both bivalve species, suggesting oxidative stress endured by the organisms, although were not observed changes at the TBARS levels under laboratorial conditions (10 µg Cu/L). Regarding other bivalves species, Zhang *et al*.^9^, did not report significant changes in the GST and GPx activities to *Chlamys farreri* exposed to 3 µg Cu /L, during 96 hours. However, according to De Almeida *et al*.^50^, when *Perna perna* was exposed to 40 µg/L of copper sulphate, during 120 hours, after 72 hours of exposure was observed a depletion of GSH levels, despite GPx and GST not exhibiting significant changes. Moreover, Canesi *et al*.^51^ also reported a decrease of the GSH levels on *Mytilus galloprovincialis* exposed to 40 µg Cu/L after 72 hours. According to the above cited studies, it is often observed a decrease of GSH levels, indicating a possible effect of the metal exposure. Despite the antioxidant enzymes do not exhibit significant changes, this absence of significant effects may be due to the low concentrations used in previous studies. Moreover, different tissues may exhibit different responses to metal exposure^3,52^. Considering longer exposure times, Maria and Bebianno (2011)^3^ observed a significant increase of LPO on *Mytilus galloprovincialis* up to 200% in the gills. The lipid peroxidation in *M. galloprovincialis* was also observed after 6 days at concentration of 40 µg Cu/L^53,54^. Furthermore, to *Ruditapes decussatus* exposed to 2.5 µg Cu/L after 28 days was also reported an increase of LPO^55^.

## Conclusions

The present work underlines the copper sulphate effects at both size classes of the bivalves’ species tested, mainly the big size class of *C. edule* at the first concentration of the chemical (0.35 mg/L). This study highlights the antioxidant enzymatic activities as good biomarkers to detect the effects of metals, as already studied by several researchers, in this case with particular emphasis to copper sulphate presence in aquatic systems. Furthermore, the present work also reveals muscle (foot) as a good tissue to determine changes in the activity of antioxidant enzymes and the occurrence of lipid peroxidation. Although it is not usual the use of this tissue to evaluate the effects on the antioxidant system, with the gills and the digestive gland being the tissues commonly used for this analysis due to the higher activity of the antioxidant enzymes in these tissues, the muscle may store greater lipid content. Thus, it may be established a closer relationship between the changes of the antioxidant enzymes activity, lipid peroxidation and alterations on the lipid profile in the muscle tissue to assess the biochemical effects of pollutants in bivalve species. Moreover, since the foot also allow us to understand the effects at enzymatic level is possible to use this tissue as an alternative to the whole organism or visceral mass, since the tissue amount is much lower and at logistic and economic levels, the analysis are easiest and less expensive. Studies evaluating the sub-lethal effects of this metal on the ecosystems and biological organisms have a great importance because it generates knowledge to act earlier in situations of exposure to the pollutants.

## Supplementary information

Supplementary information 1
