# Supplementary material for: Copper sulphate impact on the antioxidant defence system of the marine bivalves Cerastoderma edule and Scrobicularia plana
Source: Sci Rep. 2019 Nov 11;9:16458. doi: 10.1038/s41598-019-52925-9 (PMC6848077; doi:10.1038/s41598-019-52925-9)
Supplement: Supplementary file 1 — Supplementary information 1 [file 41598_2019_52925_MOESM1_ESM.docx]

**Copper sulphate impact on the antioxidant defence system of the marine bivalves *Cerastoderma edule* and *Scrobicularia plana***

Andreia F. Mesquita^a^, Sérgio M. Marques^a,^, João C. Marques^b^, Fernando J. M. Gonçalves^a^, Ana M. M. Gonçalves^a,b,*^

^a^Department of Biology and CESAM, University of Aveiro, 3810-193 Aveiro, Portugal

^b^MARE - Marine and Environmental Sciences Centre, Department of Life Sciences, Faculty of Sciences and Technology, University of Coimbra, 3004-517 Coimbra, Portugal

*Corresponding author: amgoncalves@uc.pt

***In:* Fatty acids proﬁles modiﬁcations in the bivalves *Cerastoderma edule* and *Scrobicularia plana* in response to copper sulphate. Ecological Indicators 85: 318-328.**

**Authors:** A.F. Mesquita^a^, F. Gonçalves^a^, T. Verdelhos^b^, J.C. Marques^b^, A.M.M. Gonçalves^a,b,⁎^

^a^ Department of Biology and CESAM, University of Aveiro, 3810-193 Aveiro, Portugal

^b^ MARE (Marine and Environmental Sciences Centre), Faculty of Sciences and Technology, University of Coimbra, 3004-517 Coimbra, Portugal

*corresponding author: A. M. M. Gonçalves; e-mail: amgoncalves@uc.pt

**Supplementary information 1:** Values of lethal concentration (LC) of copper sulphate for *S. plana* and *C. edule* (big and small sizes classes). In brackets are indicated the 95% confidence limits

| *Scrobicularia plana* |  | Big size (mg/L) | Small size (mg/L) |
| --- | --- | --- | --- |
|  | LC_10_ | 1.456 (0.699; 1.860) | 1.238 (0.000; 2.175) |
|  | LC_20_ | 1.836 (1.265; 2.177) | 2.428 (0.794; 3.648) |
|  | LC_50_ | 2.563 (2.229; 2.903) | 4.705 (3.540; 12.292) |
| *Cerastoderma edule* |  | Big size (mg/L) | Small size (mg/L) |
|  | LC_10_ | 0.341 (0.000; 0.571) | 0.717 (0.351; 0.895) |
|  | LC_20_ | 0.504 (0.083; 0.698) | 0.859 (0.581; 1.012) |
|  | LC_50_ | 0.818 (0.595; 0.987) | 1.129 (0.968; 1.289) |
